# *De Novo* Hepatocellular Carcinoma in Hepatitis C-Related Cirrhosis: Are Advanced Glycation End Products a Key Driver?

**DOI:** 10.3389/fcimb.2021.662431

**Published:** 2021-10-01

**Authors:** Ahmed Abdel-Razik, Walaa Shabana, Ahmed Mohamed El Nakib, Mostafa Abdelsalam, Ahmed Abdelwahab, Ahmad S. Hasan, Rasha Elzehery, Rania Elhelaly, Aya Ahmed Fathy, Sally Abdallah Mostafa, Niveen El-Wakeel, Dalia Moemen, Waleed Eldars, Ahmed H. Yassen

**Affiliations:** ^1^ Tropical Medicine Department, Faculty of Medicine, Mansoura University, Mansoura City, Egypt; ^2^ Nephrology and Dialysis Unit, Internal Medicine Department, Faculty of Medicine, Mansoura University, Mansoura City, Egypt; ^3^ Clinical Pathology Department, Faculty of Medicine, Mansoura University, Mansoura City, Egypt; ^4^ Public Health and Community Department, Faculty of Medicine, Mansoura University, Mansoura City, Egypt; ^5^ Medical Biochemistry and Molecular Biology Department, Faculty of Medicine, Mansoura University, Mansoura City, Egypt; ^6^ Medical Microbiology and Immunology Department, Faculty of Medicine, Mansoura University, Mansoura City, Egypt

**Keywords:** hepatocellular carcinoma, direct-acting antivirals, hepatitis C virus, advanced glycation end products, liver cirrhosis

## Abstract

**Background and Purpose:**

The advanced glycation end products (AGEs) have been implicated in different diseases’ pathogenesis, but their role in hepatocellular carcinoma (HCC) is still a matter of debate. This study aims to investigate the association of AGEs with HCC development in patients with hepatitis C-related cirrhosis.

**Methods:**

Only 153 of the 181 non-diabetic patients with cirrhosis were consecutively involved in this pilot cohort prospective study, along with 34 healthy control participants. Demographic characteristics, biochemical parameters, clinical data, and AGEs levels in all subjects at the starting point and every year after that for two years were assessed. Multivariable Cox regression analysis was used to settle variables that could predict HCC development within this period.

**Results:**

HCC developed in 13 (8.5%) patients. Univariate Cox regression analysis reported that body mass index (P=0.013), homeostatic model assessment-insulin resistance (P=0.006), alpha-fetoprotein (P <0.001), and AGEs levels (P <0.001) were related to HCC development. After adjusting multiple confounders, the multivariable Cox regression model has revealed that AFP and AGEs were the powerful parameters related to the HCC occurrence (all P<0.05). AGEs at a cutoff value of more than 79.6 ng/ml had 100% sensitivity, 96.4% specificity, and 0.999 area under the curve (all P<0.001), using the receiver operating characteristic curve, for prediction of HCC development.

**Conclusion:**

This work suggests that AGEs are associated with an increased incidence of HCC, particularly in cirrhosis, which is encouraging in decreasing the risk of HCC in these patients.

## Introduction

Chronic hepatitis C virus (HCV) infection is a crucial factor in the etiology of hepatic fibrosis. HCV is a major environmental problem and the leading cause of chronic liver injury and neoplastic transformation. It is one of the most frequently stated problems with liver cancer and cirrhosis (Perz et al., 2006).

Hepatocellular carcinoma (HCC) is one of the most widespread cancers and causes tumor-related mortality (Colombo and Sangiovanni, 2015). That is why chronic liver disease in many HCC patients results from HCV or hepatitis B virus (HBV) infections (Perz et al., 2006). Since the detailed pathogenetic mechanisms of HCC remain unclear, several studies are investigating new pathways (Ružić et al., 2018).

Advanced glycation end products (AGEs), a heterogeneous cluster of irreversible reactive end products created by oxidation of lipids and proteins, and non-enzymatic glycation, have been embroiled in the pathogenesis of numerous disorders (Marta et al., 2004; Zhou et al., 2004; Hyogo et al., 2007; Piarulli et al., 2013; Abdel-Razik et al., 2020a) because they stimulate the oxidative stress generation and consequently induce inflammation (Yamagishi et al., 2012).

There is accumulating evidence to display that AGEs participate in the pathogenesis of various disorders such as alcoholic liver injury, diabetic vascular complications, Alzheimer’s disease, osteoporosis, non-alcoholic steatohepatitis (NASH), cancer growth, and metastasis (Hyogo and Yamagishi, 2008; Takeuchi and Yamagishi, 2008; Takino et al., 2012; Hayashi et al., 2013; Takeuchi et al., 2015).

As mentioned in the literature, there is a great deal of attention to the role of AGEs in cancer progression and initiation. Several reports have shown that AGEs have an attainable function in cancer invasion, migration, proliferation, and survival in prostate, lung cancer cell lines, and breast (Takino et al., 2010; Rodriguez-Teja et al., 2015; Sharaf et al., 2015). To date, far too little attention is paid to human researches. The higher rate of AGEs accretion is described in cancerous lesions than benign tissues (Palimeri et al., 2015).

These remarks lead us to hypothesize that AGEs could also participate in HCC pathogenesis, particularly in those with cirrhosis. To date, there is almost no evidence associating AGEs with HCC occurrence. This study explores the relationship of AGEs with the development of HCC in patients with hepatitis C-related cirrhosis.

## Patients and Methods

This cohort pilot single-center prospective study was accomplished at the Department of Tropical Medicine (Mansoura University-Egypt) between September 2015 and November 2019. We registered 299 consecutive patients with liver cirrhosis examined in our department. Only 181 patients fulfilled the inclusion criteria and were involved in this study. Patients’ hematological, biochemical, clinical, and demographic data were appraised at the starting point and during this study.

Criteria for selecting the subjects were as follows; patients whom 1) aged ≥18 years, and 2) had liver cirrhosis.

The exclusion criteria were 1) liver cirrhosis due to autoimmune hepatitis, HBV, NASH, and cholestatic or metabolic liver diseases, 2) alcoholic liver disease, 3) lactation and pregnancy, 4) hematologic disorders and kidney diseases; 5) pancreatitis, 6) diabetes mellitus, 7) impaired glucose tolerance, 8) smoking, 9) peritoneal carcinomatosis, 10) osteoporosis, 11) abdominal tuberculosis, 12) uncontrolled thyroid disorders, 13) cancers including HCC at baseline 14) bone marrow suppression, 15) collagen vascular diseases, 16) heart failure, 17) Alzheimer’s disease or cerebrovascular accident, 18) immunosuppressive agents administration, 19) missing patient’s data, and 20) usage of anticoagulant or antiplatelet treatment, oral contraceptive drugs, hepatotoxic drugs, and NSAIDs.

The control group involved 34 healthy control subjects who were age- and sex-matched (female/male = 12/22).

The starting point records were gathered within seven days from the time of registration. The end-of-study records were picked up in the last seven days of the first and second follow-up years. By the end of the study, biochemical results, radiological findings, and clinical examination of the patients did not display any troubles that were not documented at the onset of this work affecting the follow-up variables in the subjects’ fitness.

### Diagnosis of Liver Cirrhosis and Its Complications

We assessed liver cirrhosis by unequivocal biochemical results, clinical assessment, elastography, histopathological evaluation of hepatic tissue, abdominal ultrasonography (US), or endoscopic findings suggestive of portal hypertension plus stigmata of chronic liver disease (Abdel-Razik et al., 2020b). The severity of cirrhosis was evaluated based on the Model For End-Stage Liver Disease (MELD) scoring system (Kamath et al., 2001) and the Child-Turcotte Pugh (CTP) classification.

Complications of liver cirrhosis were managed according to the standardized therapeutic methods during the study period (Cardenas and Ginès, 2005).

### Diagnosis of HCC

In all patients with cirrhosis, alpha-fetoprotein (AFP) and US- which are considered noninvasive diagnostic tools, especially for earlier stages of HCC- were performed every 3-6 months for surveillance purposes. HCC was confirmed if two imaging instances showed a focal hepatic lesion >2 cm in diameter with highlights of arterial hypervascularization or solitary radiologic finding with these highlights conjoined with a serum AFP level of >400 ng/ml (Wang et al., 2015). Management and surveillance of individuals with HCC were achieved according to clinical practice guidelines for hepatocellular carcinoma (Colombo and Sangiovanni, 2015).

### Treatment

According to EASL recommendations (EASL, 2015), individuals with CTP-B and CTP-A cirrhosis, either naive patients or treatment-experienced- who had not attained sustained virologic response (SVR) after being treated with pegylated interferon (Peg IFN)/Sofosbuvir (SOF) or with Peg IFN and ribavirin (RBV)- or those treated with RBV/SOF only, were treated based on the study practice.

This study protocol was designed according to EASL guidelines for therapy of HCV, 2015 (EASL, 2015). Patients infected with HCV (genotype IV) received therapy with DCV (60 mg) and SOF (400 mg) once daily for 12 weeks, in addition to RBV daily according to their weight, i.e., 1200 mg for patients weighing ≥75 kg or 1000 mg for patients weighing <75 kg. The dose of RBV was adjusted according to the estimated glomerular filtration rate (eGFR) (El-Khayat et al., 2018). In subjects with contraindications to the use of RBV, the duration of therapy was extended to 24 weeks (EASL, 2015).

Treatment-experienced individuals were retreated with a combination of DCV plus SOF for 24 weeks without RBV or 12 weeks with RBV (EASL, 2015).

### Monitoring of Treatment Efficacy

HCV RNA level was assessed at baseline, at the end of therapy (12 or 24 weeks), and 12 weeks after finishing therapy using the Roche COBAS Taq Man HCV assay version 2.0 (detection limit is 15 I.U./mL). Relapse is defined as detectable HCV RNA level during follow-up in a patient with a previously undetected level at the end of the therapy, and virological failure is referred to as nonresponse where HCV RNA levels are still detected at the end of the therapy. The primary virological response was the accomplishment of SVR12, described as the concentration in serum lower than 15 I.U./mL (El-Khayat et al., 2018).

The levels of AGEs were measured at the starting point and 12 weeks after finishing therapy.

### Data Collection

Qualified examiners congregated data. To identify these data, they asked the participants to finish a standardized, self-validated questionnaire. These queries have provided us with info about employment, marital status, alcohol consumption, and medical history, particularly malignancy, diabetes, and smoking habits, alongside medication history. Body mass index (BMI) was calculated as weight (kg) divided by the square of height (m^2^).

### Sampling

Fresh venous blood samples (6mL) were collected from all subjects after overnight fasting (4mL with no anticoagulants for serum testing and 2mL on EDTA for hemogram). The mixture was centrifuged for 10 min at 1000-3000 RPM. The serum samples were then divided into aliquots and frozen at −20°C until subsequent estimations.

### Methodology

Routine blood tests, including complete blood count (CBC), liver function tests, serum creatinine, serum cholesterol and triglycerides were performed.

Serum AFP was evaluated by chemiluminescent immunometric technique on the Immulite 2000 system (Siemens Medical Solutions Diagnostics, Los Angeles, California, USA). The eGFR was estimated based on the following formula: $eGFR=186\times {\left(\frac{Creatinine}{88.4}\right)}^{-1.154}\times {Age}^{-0.203}\times \left(1.210\;if\;black\right)\times \left(0.742\;if\;female\right)$ (Levey et al., 1999). Quantitative assessments of serum AGEs were measured by ELISA kit (MyBioSource, San Diego, CA 92195-3308, USA, Cat No. MBS267540). The homeostasis model assessment (HOMA) method (Matthews et al., 1985) was evaluated as follows: Insulin resistance (HOMA-IR) = $\frac{fasting\;serum\;insulin\;(FSI)\times fasting\;plasma\;glucose\;(FPG)}{405}$

### Detection of HCV Genotype IV

Hepatitis C viral RNA was measured in patients’ sera by QIAamp Viral RNA Mini Kit (Qiagen, Hilden, Germany) (Ohno et al., 1997).

### Ethics

Mansoura Institutional Review Board reviewed and accepted all procedures in this study protocol. Moreover, all participants gave informed consent before the onset of this study. This work was held according to the Helsinki Declaration’s rules. (Proposal Code: R.20.05.844.1R1).

### Statistical Analysis

Data management and analysis were performed using the Social Package of Statistical Science (SPSS) software version 20 (SPSS Inc., Chicago, IL, USA). Quantitative data and non-normally distributed continuous data are designated as mean ± SD and Range or Interquartile range correspondingly. Student t-test, Mann-Whitney U test, and Chi-square test were used for normally distributed data, non-normally distributed continuous data, and categorical data, correspondingly. The relation between AGEs level and other parameters was executed by Spearman’s correlation analysis. At univariate analysis, parameters with a *P* value less than 0.05 were combined in the multivariable Cox regression analysis. Multivariable and univariate Cox regression models were calculated to reveal the independent factors related to predicting HCC. The area under the curve (AUC) and receiver operating characteristic curve (ROC) were applied, and the best cutoff values were estimated to expect the development of HCC. A two-tailed *P* value of less than 0.05 was considered significant.

## Results

### Patient Characteristics

**Figure 1:**
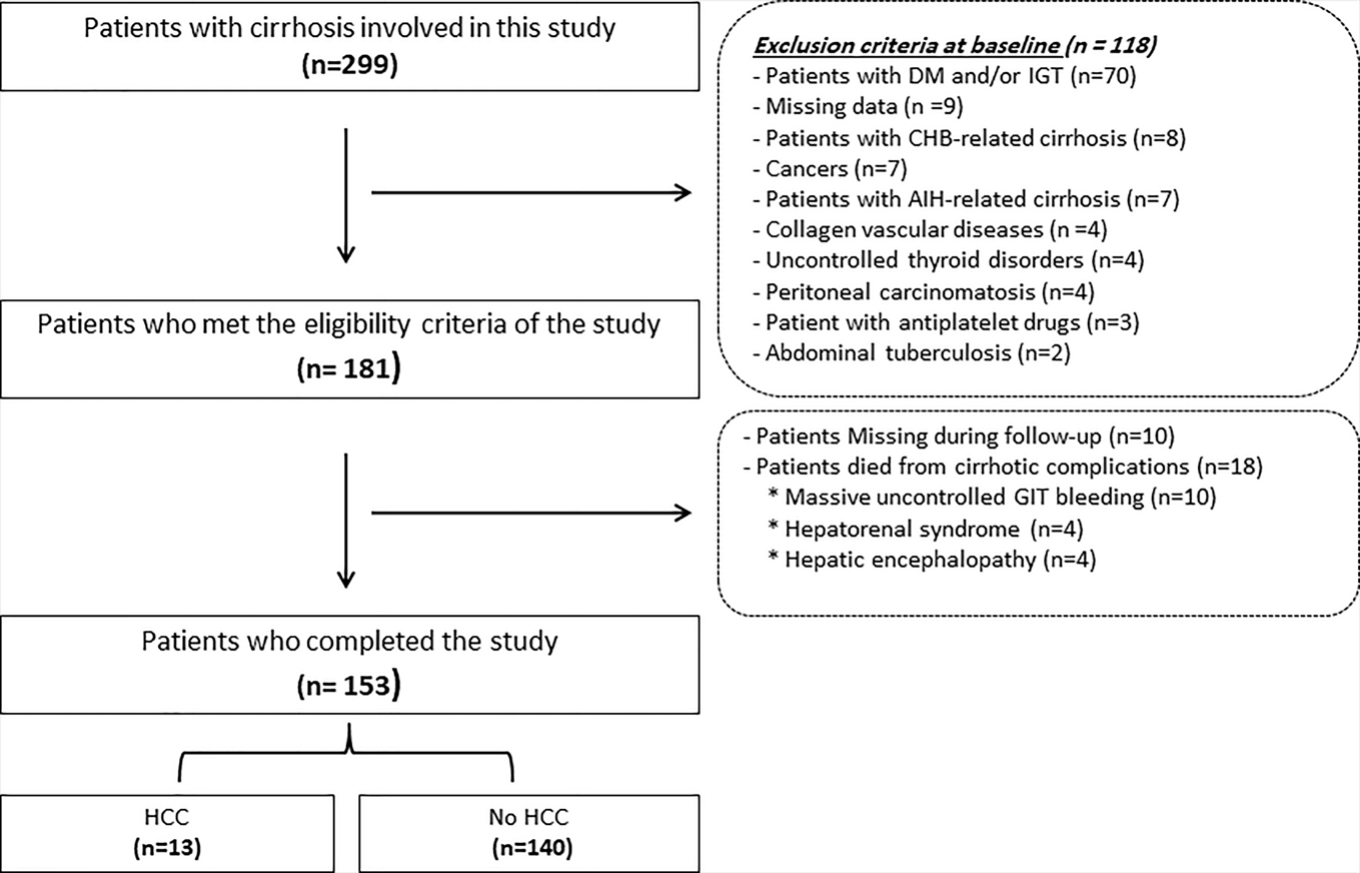
Flowchart of the patients included in this study. HCC, hepatocellular; DM, diabetes mellitus; AIH, autoimmune hepatitis; GIT, gastrointestinal tract; CHB, chronic hepatitis B.

One hundred fifty-three patients joined this study out of the one hundred eighty-one who fulfilled the inclusion criteria and were initially registered. Eighteen out of the involved one hundred eighty-one patients in this study died from complications of cirrhosis, e.g., hepatic encephalopathy (n=4), massive uncontrolled GIT bleeding (n=10), and hepatorenal syndrome (n=4), and we lost ten patients during the follow-up period as displayed in **Figure 1**. **Table 1** provides the results obtained from the preliminary analysis of the enrolled individuals’ clinical, demographic, and biochemical characteristics at baseline.

**Table 1:** Baseline biochemical, demographic, and clinical characteristics of enrolled participants.

Characteristics	Patients with cirrhosis (*n* = 153)	Control group (*n* = 34)	*P* Value
Age (year)	57.81 ± 5.4	56.4 ± 4.2	0.15
Sex (male/female)	99/54	22/12	0.57
Body mass index (kg/m^2^)	26 ± 1.05	25.70 ± 0.34	0.1
Patients receiving DAA (n=117)			
SVR	87 (74.4)	–	–
Non-responder	30 (25.6)	–	–
Patients not receiving DAA	36	–	–
Hb (gm/dl)	9.14 ± 0.71	12.06 ± 0.35	<0.001
WBCs (x10^3^/cm^2^)	3.5 (3.2-3.8)	6.1 (5.25-7.5)	<0.001
Platelet count (×10^3^/cm^2^)	80.49 ± 24.2	202.53 ± 50.92	<0.001
Total cholesterol (mg/dl)	177.88 ± 13.6	174.12 ± 10.1	0.13
Triglyceride (mg/dl)	123.28 ± 16.16	128.24 ± 7.8	0.08
AST (U/l)	42 (30-52)	31 (22-33.25)	<0.001
ALT (U/l)	38 (28-49)	28 (25.-31.2)	<0.001
GGT (U/l)	38.63 ± 10.84	22.17 ± 4.7	<0.001
ALP (IU/ml)	100 (93.0-110.0)	53 (44.7-65.2)	<0.001
Bilirubin (mg/dl)	2.1 (1.6-2.85)	0.9 (0.77-1.03)	<0.001
Albumin (g/dl)	2.99 ± 0.42	4.17 ± 0.18	<0.001
INR	1.56 ± 0.27	0.93 ± .08	<0.001
Creatinine (mg/dl)	1.22 ± 0.27	0.89 ± 0.16	<0.001
eGFR	76.3 ± 9.4	96.3 ± 6.2	<0.001
Child-Pugh score	36 (24)	–	–
	52 (34)	–	–
	65 (42)	–	–
MELD score	17.03 ± 4.05	–	–
FPG (mg/dl)	88.27 ± 7.6	87.88 ± 4.9	0.78
HOMA-IR	3.7 (3.4-4.2)	1.8 (1.6-2)	<0.001
AFP (ng/ml)	45 (23-66)	7 (5.7-9)	<0.001
AGEs (ng/ml)	73.2 ± 19.6	14.3 ± 1.4	0.001

Data were presented as mean ± SD, median and interquartile range or n (%).

SVR, sustained virological response; DAA, direct acting antiviral; AST, aspartate aminotransferase; HOMA-IR, homeostasis modelAssessment-insulin resistance, INR, international normalized ratio; FPG, fasting plasma glucose; AGEs, advanced glycation end products; ALP, alkaline phosphatase; eGFR, estimated glomerular filtration rate; WBCs, white blood cells; MELD, Model for End-Stage Liver Disease; GGT, γ-glutamyl transpeptidase; ALT, alanine aminotransferase.

Patients displayed a statistically significant increase in AST, ALP, ALT, GGT, serum creatinine, serum bilirubin, HOMA-IR, INR, AFP, and AGEs compared to healthy subjects (all p<0.05), and a statistically significant decrease in WBCs, hemoglobin, serum albumin, eGFR, and platelet count compared to healthy subjects (p<0.05).

After the primary assessment at baseline by biochemical and hematological blood tests, the participants were followed up every year for two years.

**Figure 2:**
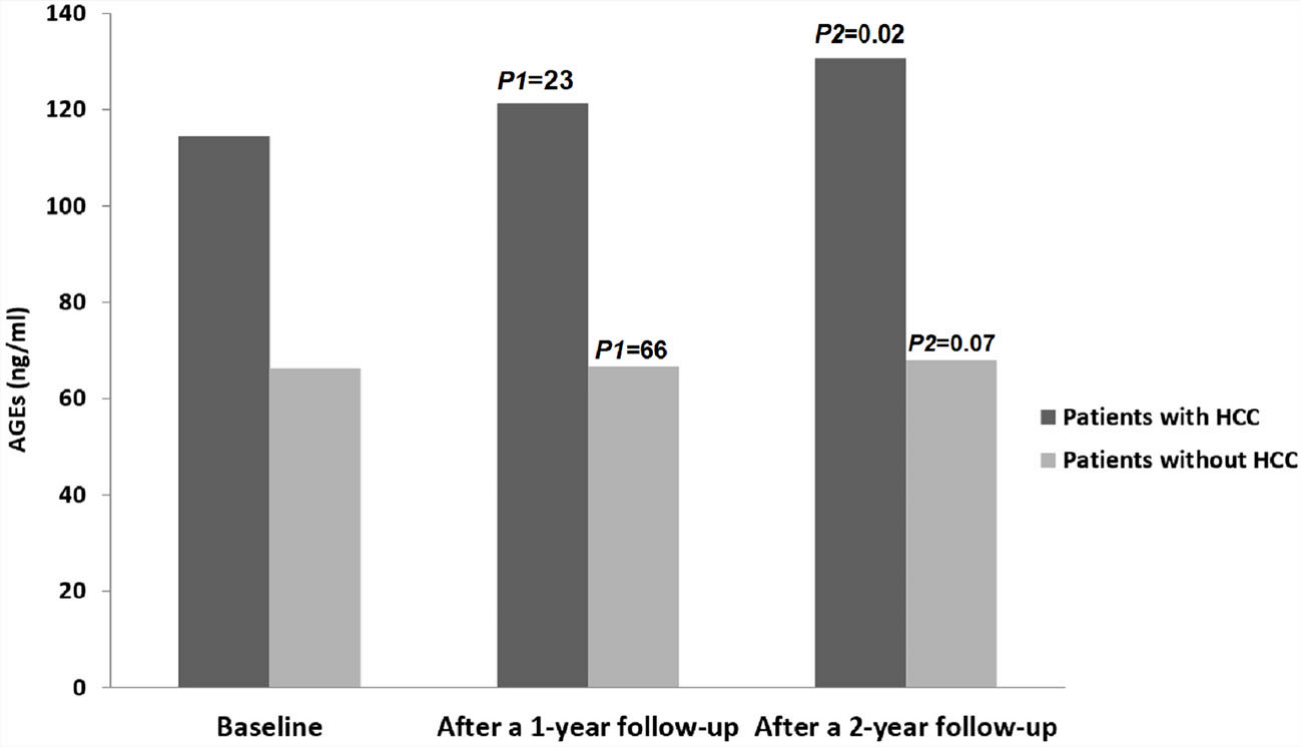
Levels of AGEs in all patients throughout the study period. HCC, hepatocellular; AGEs, Advanced glycation end products. P1 = Baseline vs. after 1 year follow-up; P2 = Baseline vs. after 2 year follow-up.

Out of all the patients assessed during the follow-up period (n=153), thirteen patients (8.5%) developed HCC. **Figure 2** shows serum levels of AGEs in patients with HCC compared to patients without HCC.

Regarding the control group, there were no statistically significant differences in the levels of AGEs between the starting point and at the end-of-study (15.3 ± 2.2 vs. 16.2 ± 2.3; *P*=0.09), and none of them developed HCC during the follow-up period.

### Correlation Between Biochemical Parameters and AGEs in the Studied Patients

**Figure 3:**
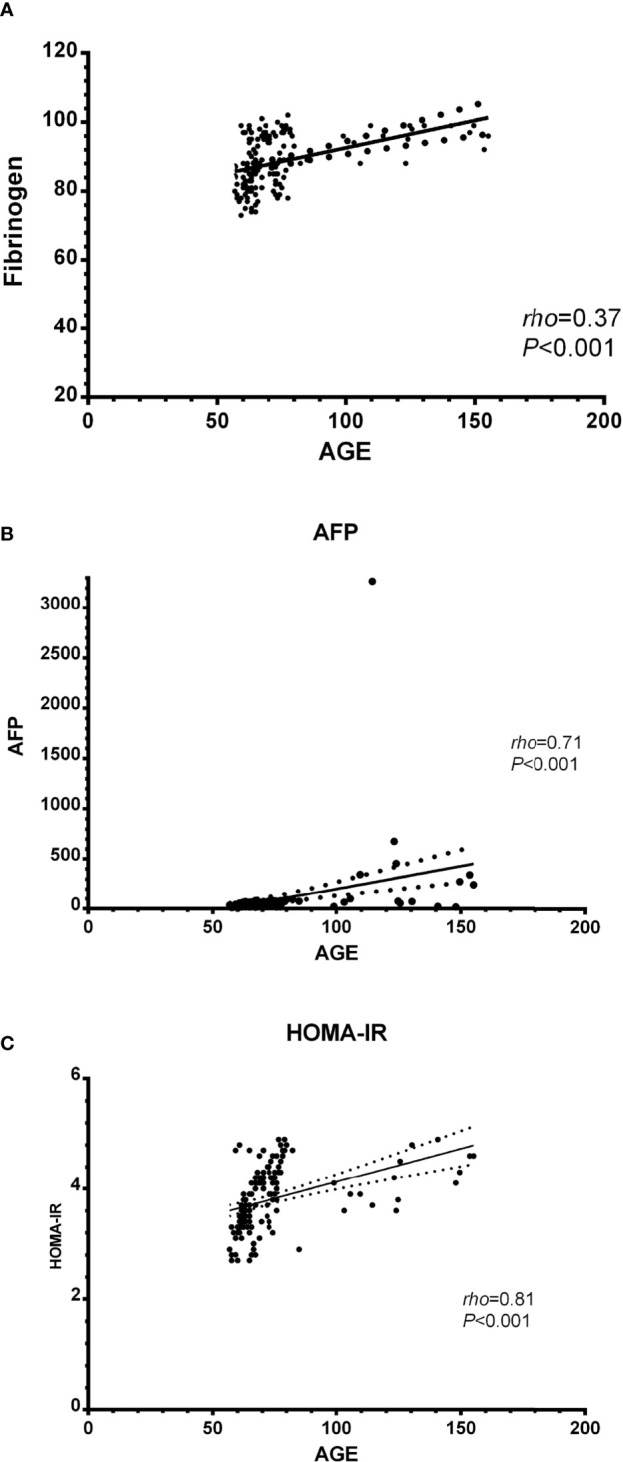
Correlation between biochemical parameters and AGEs in the studied patients; **(A)** AGEs and fibrinogen, **(B)** AGEs and AFP, and **(C)** AGEs and HOMA-IR. AGEs, Advanced glycation end products; AFP, alpha-fetoprotein; HOMA-IR, Homeostatic model assessment-insulin resistance.

Spearman’s correlation analysis revealed there was a significant positive correlation between serum AGEs and fibrinogen, AFP, and HOMA-IR (*rho*=0.37, *P*<0.001; *rho*=0.71, *P*<0.001; and *rho*=0.81, *P*<0.001 individually) see **Figure 3**.

### Multivariable and Univariate Cox Regression Models Predicting HCC Within the Two Years Follow-Up Period

**Table 2** presents an overview of the patients’ biochemical, clinical, and demographic features with and without HCC.

**Table 2:** Biochemical, demographic, and clinical characteristics of patients without and with HCC during the follow-up period.

Characteristics	No HCC (*n* = 140)	HCC (*n* = 13)	*P* value
Age (year)	57.87 ± 5.53	57.07 ± 4.19	0.613
Sex (male/female)	91/49	8/5	0.5
Body mass index (kg/m^2^)	26.7 ± 1.09	25.9 ± 1.03	0.012
Patients receiving DAA			
SVR	77 (55)	10 (77)	0.127
Non-responder	28 (20)	2 (15.3)	0.684
Patients not receiving DAA	35 (25)	1 (7.7)	0.161
Hb (gm/dl)	9.15 ± 0.65	9.14 ± 0.7	0.958
WBCs (x10^3^/cm^2^)	3.45 (3.2-3.8)	3.6 (3.15-3.75)	0.778
Platelet count (×10^3^/cm^2^)	51 (45.-65.75)	110 (57.5-117.)	<0.001
Total cholesterol (mg/dl)	177.55 ± 13.85	181.46 ± 10.3	0.323
Triglyceride (mg/dl)	122.81 ± 16.48	128.31 ± 11.48	0.242
AST (U/l)	41.5 (29.25-51)	49 (30.5-71.5)	0.02
ALT (U/l)	37.5 (28-48)	48 (27.5-66.5)	<0.001
GGT (U/l)	38.82 ± 11.025	36.53 ± 8.685	0.468
ALP (IU/ml)	100 (92-108.75)	110 (100-122)	<0.001
Bilirubin (mg/dl)	2.1 (1.6-2.8)	1.45 (2.3-3.7)	0.569
T protein	6.4 (6.2-6.6)	6.7 (6.35-6.8)	<0.001
Albumin (g/dl)	2.97 ± 0.39	3.30 ± 0.59	0.006
INR	1.55 (1.3-1.7)	1.7 (1.4-1.85)	0.704
Creatinine (mg/dl)	1.3 (1.1-1.4)	1.4 (.95-1.5)	0.695
eGFR	78.8 ± 9.6	75.4 ± 9.2	0.222
Child-Pugh score			0.816
A	32 (22.9%)	4 (31%)	
B	47 (33.6%)	5 (38%)	
C	61 (43.5%)	4 (31%)	
MELD score	16.9 ± 3.89	17.84 ± 5.58	0.425
FPG (mg/dl)	87.521 ± 7.50	96.3 ± 3.27	<0.001
HOMA-IR	3.76 ± 0.55	4.24 ± 0.42	0.003
AFP (ng/ml)	24.3 (23-63)	44 (39 -400)	<0.001
AGEs (ng/ml)	67.96 ± 7.71	130.6 ± 17.8	<0.001

Data were presented as mean ± SD, median and interquartile range or n (%).

SVR, sustained virological response; DAA, direct acting antiviral; AST, aspartate aminotransferase; HOMA-IR, homeostasis modelAssessment-insulin resistance, INR, international normalized ratio; FPG, fasting plasma glucose; AGEs, advanced glycation end products; ALP, alkaline phosphatase; eGFR, estimated glomerular filtration rate; WBCs, white blood cells; MELD, Model for End-Stage Liver Disease; GGT, γ-glutamyl transpeptidase; ALT, alanine aminotransferase.

There was no significant difference in total cholesterol, hemoglobin, sex, age, WBCs, serum triglyceride, GGT, INR, serum bilirubin, serum creatinine, CTP classification, MELD score, and eGFR between the two groups (all *P*>0.05). Univariate Cox regression analysis showed that BMI, HOMA-IR, AFP, and AGEs levels are statistically significant between the two groups (all *P*<0.05) in **Table 3**.

**Table 3:** Cox regression analysis models to predict the development of HCC in the studied patients.

Variables	Univariate Cox regression			Multivariable Cox regression		
	HR	95% CI	*P* value	HR	95% CI	*P* value
BMI	1.846	1.141 - 2.985	0.013	1.807	0.875-3.733	0.112
HOMA-IR	4.5562	1.558- 13.324	0.006	0.5200	0.106- 2.543	0.422
AFP	1.0014	1.0008-1.0021	<0.001	1.0014	1.0006- 1.0023	0.001
AGE	1.074	1.051-1.098	<0.001	1.082	1.049-1.115	<0.001

BMI, Body mass index; HOMA-IR, Homeostatic model assessment- insulin resistance; AFP, alpha-fetoprotein; AGEs, advanced glycation end products; HR, hazard ratio.

The multivariable Cox regression analysis model outcomes were reassessed after adjusting multiple confounders, applying the formerly described variables at baseline associated with the development of HCC during the two-year follow-up period. This test showed that the only parameters independently related to the development of HCC are AFP and AGEs (**Table 3**).

**Figure 4:**
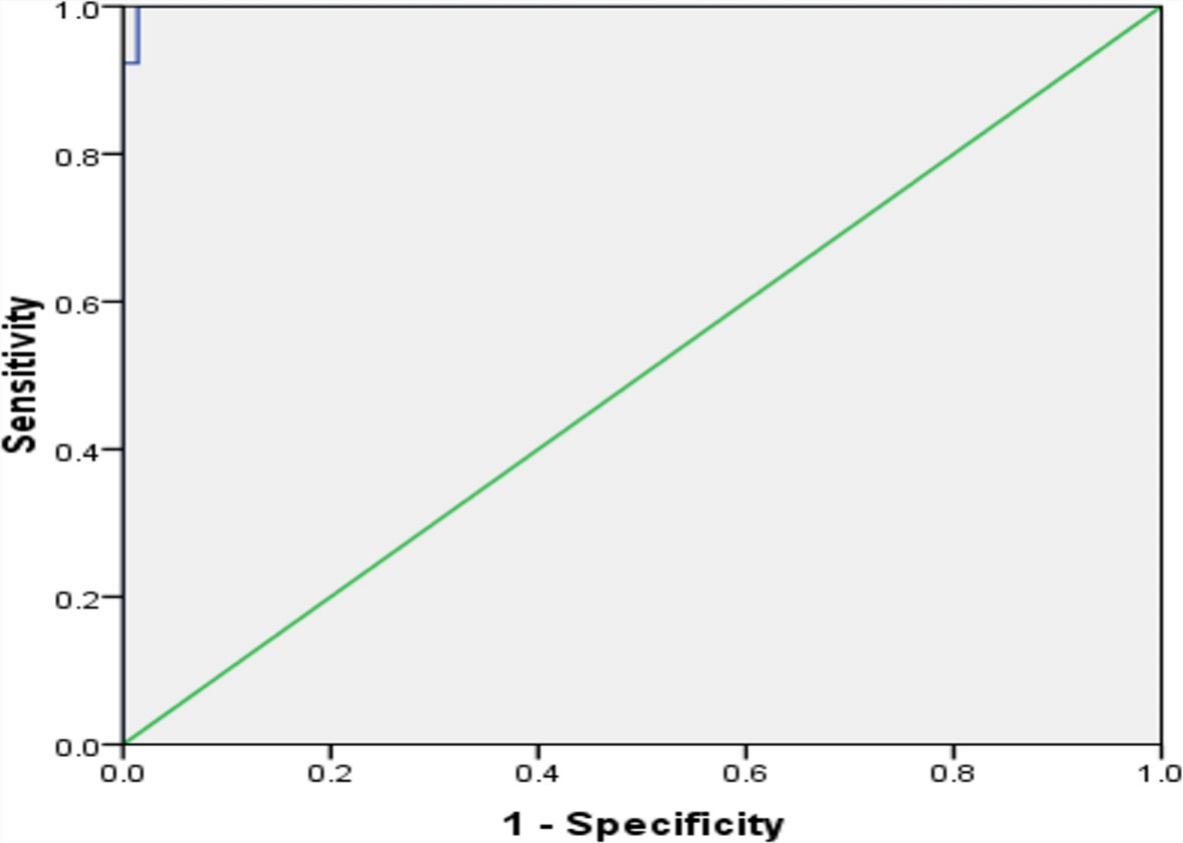
The receiver operating characteristic curve of AGEs in predicting HCC in patients with cirrhosis.

Using the ROC curve, at a cutoff value of > 79.6 ng/ml, AGEs had 100% sensitivity, 96.4% specificity, 0.999 AUC, 100% negative predictive value, 72.2% positive predictive value, and *P*<0.001 for prediction of HCC, as presented in **Figure 4**.

All patients were followed-up regularly (mean 26 ± 6 months) to check the different therapy modalities’ effectiveness for HCC. Regarding AGEs, there was a significant reduction of serum AGE at baseline and 3-months after therapy as follows (130.6 ± 17.7 vs. 66.84 ± 7.68; *P*<0.001).

### Therapeutic Findings

According to our study protocol, participants with CTP-B (n=52) and CTP-A (n=65) cirrhosis received treatment. SVR showed in 87 (74.4%) patients. No significant difference was detected in AGEs after completion of therapy vs. at baseline (73.8 ± 20.8 vs. 78.6 ± 21.4; P=0.1).

AGEs presented no significant differences at starting point and at the end of treatment regarding relapse (n=11) (76.5 ± 20.2 vs. 80.6 ± 21.2; P=0.647), non-responder (n=19) (78.6 ± 21.5 vs. 82.1 ± 21.8; P=0.621), and SVR (n=87) (75.6 ± 21.3 vs. 80.2 ± 21.6; P=0.159).

There was no statistically significant change between patients who did not receive treatment and those who did (1/36 vs. 12/117; *P*= 0.161) correspondingly regarding the development of HCC.

### Safety and Tolerability

The most popular side effects were pruritus (9.2% and 13.5%), headache (7.7% and 11.5%), hyperbilirubinemia (12.3% and 19.2%), RBV dose reductions (10.8% and 17.3%), anaemia (15.4% and 25%), and fatigue (13.8% and 27%) in CTP-A and CTP-B patients; correspondingly.

We registered critical adverse events in CTP-B cirrhotic patients more than CTPA; (6.2% and 9.6%) had GIT bleeding, (4.6% and 7.7%) had hepatic encephalopathy, (7.7% and 11.5%) developed ascites, (7.7% and 13.5%) had HCC, and (1.5% and 1.9%), had renal impairment in CTP-A and CTP-B respectively.

## Discussion

The interaction of the receptor for advanced glycation end products (RAGE) with AGEs is believed to cause chronic pathophysiology through various processes (Tabrez et al., 2015). The relationship between the development of HCC and RAGE activation achieved consideration as a consequence of the liver’s role in the metabolism of AGE (Su et al., 2015). Experimental reports also proposed the RAGE-AGE axis role in initiating different hepatic disorders and tumorigenesis (Hyogo and Yamagishi, 2008; Hiwatashi et al., 2008).

Our patients’ most significant finding was that increased AGEs levels could be deemed an independent variable for predicting HCC occurrence.

AGEs-provoked reactive oxygen species (ROS) release might intercede *via* nuclear factor-kappa (NF-κB) independent and/or dependent manners. Stimulated NF-κB initiates tumor necrosis factor-alpha (TNF-α) release, which unites to tumor necrosis factor receptor 1 (TNFR-1) and then produces ROS (Neumann et al., 1999; Janssen-Heininger et al., 2000), which in turn run in a vicious series of TNF-α and ROS production. Numerous further intracellular signaling molecules, such as stress-activated protein kinase/c-Jun N-terminal kinase, mitogen-activated protein kinases (MAPK), as p38 extracellular signal-regulated kinase-1 are included in the AGE-stimulated TNF-α production and ROS release (Requena et al., 2009). These abnormalities can provoke the progression and development of HCC (Muriel, 2009; Zhao et al., 2015).

Furthermore, DNA’s oxidative injuries lead to mutagenic and carcinogenic effects by dividing cells with unpaired or mis-repaired injuries, which finally provoke the development and progression of HCC (Yang et al., 2014).

There is a striking observation, which is a sharp decline in serum AGEs after therapy for HCC. This may perhaps elucidate the role of RAGE in the pathogenesis of HCC, especially in altering carcinogenic signaling in the cancer microenvironment (Takino et al., 2010; Yaser et al., 2012; Pusterla et al., 2013). Takino et al., 2010 issued that anti-RAGE antibody therapy can prevent cancer development, protract survival rates, and suppress spontaneous lung metastases. Hepatic tissue samples of primary HCC patients detected markedly elevated levels of RAGE mRNA. This explains the possible role of RAGE in HCC propagation (Yaser et al., 2012). Chen et al., 2014 recorded repeatedly amplified expressions of RAGE, high mobility group box-1 (HMGB1), and extracellular HMGB in keeping with cell metastasis potentials in various HCC cell lines. The RAGE-HMGB1 axis encourages cell division, immigration, and incursion and raises the level of NF-κB in HCC cell lines (Chen et al., 2014). Additionally, the precise downstream signaling ways of the RAGE-HMGB1 axis in the evolution of HCC calls for additional studies. A decrease in RAGE holds up the onset of malignant conversion in the double knockout HCC mice model, which draws attention to the role of RAGE in the development of HCC (Pikarsky et al., 2004).

In this study, there is no evidence that treatment with DAA has an influence on HCC development. In accordance with the present results, previous studies have demonstrated that DAA therapy has no impact on tumor development (Waziry et al., 2017; Gitto et al., 2021). While some other studies concluded that HCV eradication leads to decreased inflammation levels leading to decreased cancer immunosurveillance by the patient and encourages HCC development (Reig et al., 2016). To date, this problem has received scant attention in the research literature.

If HCV triggered the elevation in AGE levels (He et al., 2015), they should have gotten back to their average values after therapy with DAAs. Nonetheless, we have not detected any diminish in the estimations of their values after follow-up and treatment compared to the baseline values for these patients. This implies that cirrhosis is the main cause behind this increase.

The RAGE–AGE axis is a principal etiology in the activation of HSC and the resulting liver cirrhosis. Decreasing the RAGE–AGE signaling by avoiding overcooked foods, sensitizing insulin function, controlling high glucose, and oxidant supplement digestion (Raffaelli et al., 2014) is an auguring approach to reducing the hazard of liver cirrhosis and HCC in CHC patients, particularly with raised AGEs.

To the best of our knowledge, this study is the first to contribute to this growing area of research by exploring the impact of AGEs in such patients. These results have significant effects on understanding how AGEs were implicated in HCC development.

To the best of our insight from the accessible results, we advocate further exploitation of inflammatory signaling cascades, MAPK, and NF-κB activation as a possible target to establish a direct linkage between glycation and HCC and study the effect of the anti-RAGE antibody treatment in HCC-related cirrhosis.

The precise mechanism of AGEs in cirrhosis still needs clarification. These findings suggest several pathways for AGEs in the pathogenesis of HCC. The findings of this study have several important implications for future practice. Further research in this field would be of great help in understanding the pathogenesis of HCC and the effect of the anti-RAGE antibody therapy in such patients.

The generalizability of these results is subject to certain limitations—first, small sample size. Second, a single-center study with a follow-up period of only two years could not reveal immunological or cytokine mediators’ levels. Third, the etiology of cirrhosis is only the hepatitis C virus. Fourth, overfitting the learning curves with high AUC was noted, and it is an abstruse drawback. Model validation is frequently applied to control and assess this problem. Conversely, these methods need someone to hold or collect a considerable amount of records for validation and are rarely utilized in research comprising human individuals, where data collection is usually extraordinarily costly (Vabalas et al., 2019). Lastly, the diagnosis of HCC depends mainly on radiological and biochemical findings, not histopathological evaluation.

In conclusion, AGEs were obviously associated with an increased incidence of HCC, particularly in patients with liver cirrhosis. This may be an implying approach to reduce the hazard of HCC and liver cirrhosis in those patients.

## Data Availability Statement

The data that support the findings of this study are available on request from the corresponding author. The data is not publicly available as they contain data that could compromise the privacy of research participants.

## Ethics Statement

The studies involving human participants were reviewed and approved by Mansoura Institutional Review Board. The patients/participants provided their written informed consent to participate in this study (Proposal Code: R.20.05.844.1R1).

## Author Contributions

AA-R, and WS contributed to the study design and concept, conducted the literature search, supervised the study, and wrote the manuscript; MA, AA, AMN and AY made the tables and figures and contributed to the data analysis; RanE, RasE, and NE-W contributed to the collection of patients' samples and medical information; as well as contributed to the analysis of data and acquisition; and contributed to the acquisition of data; AH contributed to the study concept and critically revised the manuscript; AAF, SAM, DM and WE performed the statistical analysis and contributed to the analysis of data and acquisition. All the authors have accepted responsibility for the entire content of this submitted manuscript and approved submission. All authors approved the final version of the article, including the authorship list.

## Conflict of Interest

The authors declare that the research was conducted in the absence of any commercial or financial relationships that could be construed as a potential conflict of interest.

## Publisher’s Note

All claims expressed in this article are solely those of the authors and do not necessarily represent those of their affiliated organizations, or those of the publisher, the editors and the reviewers. Any product that may be evaluated in this article, or claim that may be made by its manufacturer, is not guaranteed or endorsed by the publisher.

## Abbreviations

AGEs, advanced glycation end products; HCC, hepatocellular carcinoma; AFP, alpha-fetoprotein; DAAs, direct-acting antivirals; DM, diabetes mellitus; RAGE, Receptor for Advanced Glycation Endproducts; GIT, gastrointestinal tract; BMI, Basal metabolic index; NASH, non-alcoholic steatohepatitis; HCV, hepatitis C virus; DCV, daclatasvir; SOF, Sofosbuvir; MELD, Model for End-Stage Liver Disease; CTP, Child-Turcotte Pugh.
